# The Role of Natriuretic Peptides in the Management of Heart Failure with a Focus on the Patient with Diabetes

**DOI:** 10.3390/jcm13206225

**Published:** 2024-10-18

**Authors:** Michela Vergani, Rosa Cannistraci, Gianluca Perseghin, Stefano Ciardullo

**Affiliations:** 1 Department of Medicine and Surgery, University of Milano Bicocca, 20126 Milan, Italy; m.vergani37@campus.unimib.it (M.V.); gianluca.perseghin@policlinicodimonza.it (G.P.); 2 Department of Medicine and Rehabilitation, Policlinico di Monza, Via Modigliani 10, 20900 Monza, Italy; rosa.cannistraci@policlinicodimonza.it

**Keywords:** natriuretic peptide, NT-proBNP, BNP, heart failure, diabetes mellitus

## Abstract

Natriuretic peptides (NPs) are polypeptide hormones involved in the homeostasis of the cardiovascular system. They are produced by cardiomyocytes and regulate circulating blood volume and sodium concentration. Clinically, measurements of brain natriuretic peptide (BNP) and N-terminal pro-BNP (NT-proBNP) are recommended by international guidelines as evidence is accumulating on their usefulness. They have a high negative predictive value, and in the setting of low NPs, a diagnosis of heart failure (HF) can be safely excluded in both emergency (BNP < 100 pg/mL, NT-proBNP < 300 pg/mL) and outpatient settings (BNP < 35 pg/mL and NT-proBNP < 125 pg/mL). Moreover, the 2023 consensus from the European Society of Cardiology suggests threshold values for inclusion diagnosis. These values are also associated with increased risks of major cardiovascular events, cardiovascular mortality, and all-cause mortality whether measured in inpatient or outpatient settings. Among patients without known HF, but at high risk of developing it (e.g., in the setting of diabetes mellitus, hypertension, or atherosclerotic cardiovascular disease), NPs may be useful in stratifying cardiovascular risk, optimizing therapy, and reducing the risk of developing overt HF. In the diabetes setting, risk stratification with the use of these peptides can guide the physician to a more informed and appropriate therapeutic choice as recommended by guidelines. Notably, NP levels should be carefully interpreted in light of certain conditions that may affect their reliability, such as chronic kidney disease and obesity, as well as demographic variables, including age and sex. In conclusion, NPs are useful in the diagnosis and prognosis of HF, but they also offer advantages in the primary prevention setting.

## 1. Introduction

The natriuretic peptide system involves polypeptide hormones produced by myocardial cells, primarily aimed at regulating volemia and natremia, thus maintaining cardiovascular system homeostasis. The recognition of cardiac endocrine function dates to 1964, while a definitive characterization of natriuretic peptides (NPs) was achieved approximately two decades later [1]. These molecules exhibit pleiotropic effects and continue to be investigated, especially concerning their clinical, prognostic, and predictive implications.

Among NPs, we can distinguish atrial natriuretic peptide (ANP) and B-type natriuretic peptide (BNP), synthesized predominantly in atrial cardiomyocytes and ventricular cardiomyocytes, respectively, as well as in other extra-cardiac sites, including brain (first site where BNP was identified, hence its name), gonads, and kidneys. Both peptides are released in response to cardiac wall stress induced by volemic expansion, resulting in increased cardiac room filling pressures. Conversely, C-type natriuretic peptide, while sharing a primary structure with ANP and BNP, lacks the C-terminal portion, which is essential for natriuretic action, and it is therefore not further discussed here.

ProANP is the precursor of ANP and is predominantly stored in secretory granules within atrial cardiomyocytes. Upon release into circulation, proANP undergoes cleavage by an atrial proteolytic enzyme, yielding NT-proANP and the more represented ANP, the latter comprising 28 amino acids with a half-life of about 2–4 min. BNP, isolated after ANP, is primarily released from ventricular myocardial cells in its precursor form, proBNP. This precursor is cleaved by the proteolytic enzyme corin into BNP, consisting of 32 amino acids, and into the remaining N-terminal inactive part, NT-proBNP. BNP exhibits a longer half-life than ANP, estimated at approximately 20 min [2].

ANP and BNP exert their effects by binding to two distinct receptors: natriuretic peptide receptor A (NPRA) and natriuretic peptide receptor B (NPRB), both of which are transmembrane guanylate cyclases. Upon binding with natriuretic peptides, NPRA and NPRB become activated, leading to increased intracellular levels of cyclic guanosine monophosphate (cGMP). cGMP is considered the primary second messenger responsible for most of the physiological effects mediated by the NP system, primarily through inhibition of the renin–angiotensin–aldosterone system (RAAS). NPRC is the third natriuretic peptide receptor, similar to NPRA and NPRB in its extracellular domain, but lacking intrinsic enzymatic activity, ensuring its involvement primarily in the clearance of natriuretic peptides. NPs exhibit differing physiological effects based on their specific affinities for NPRA and NPRB. NPs influence the endothelium by suppressing endothelin secretion and modulate the nervous system both centrally, by reducing thirst sensation and vasopressin secretion, and peripherally, by inhibiting the sympathetic nervous system. The overall effects of NPs include increased diuresis and natriuresis, blood pressure reduction, anti-apoptotic and anti-fibrotic actions that protect against pathological heart remodeling prior to heart failure (HF), reduced systemic vasoconstriction, and attenuation of cardiovascular sympathetic stimulation [3].

In a healthy individual, the ratio of BNP to ANP molecules is approximately one, whereas in HF, there is a significant elevation in the ratio of circulating BNP molecules to ANP molecules, proportional to the severity of cardiac dysfunction and symptoms. For instance, in advanced HF (functional class III–IV according to the New York Heart Association classification, Table 1), the BNP/ANP ratio averages 3 to 5 [4]. Consequently, the BNP assay generally exhibits superior sensitivity and diagnostic accuracy compared to the ANP assay. Concerning peptides derived from proBNP, it is observed that in healthy individuals, the values of BNP and NT-proBNP are similar. However, in the presence of left ventricular dysfunction, NT-proBNP levels increase more prominently than BNP, with NT-proBNP concentrations being approximately four times those of BNP. This disparity in peptide concentrations is attributed to different half-lives, estimated at approximately 21 min for BNP and 70 min for NT-proBNP. Moreover, NT-proBNP demonstrates superior stability at room temperature, facilitating sample handling in the laboratory more so than BNP, which is compromised under similar conditions and subject to assay-dependent variability [5,6]. In a study involving 164 patients hospitalized for HF, both NT-proBNP and BNP levels were predictive of cardiac and all-cause mortality at a 90-day follow-up, with NT-proBNP exhibiting superior prognostic value for all-cause mortality [7]. However, available data regarding the diagnostic and prognostic superiority of NT-proBNP over BNP remain limited, necessitating further investigation. Additionally, there is no universal conversion factor for comparing values between the two peptides. According to the most recent guidelines from the American College of Cardiology–American Heart Association–Heart Failure Society of America (ACC/AHA/HFSA) published in 2022 and the European Society of Cardiology (ESC) published in 2021, NPs play a pivotal role in the diagnosis, prognostic evaluation, and management of HF, as well as in individuals at high risk of developing HF [8,9]. These guidelines recommend the use of BNP and NT-proBNP interchangeably, each with their own cutoff values. In the present review, the term “natriuretic peptides” refers to BNP and NT-proBNP, unless otherwise specified.

The ESC guidelines also mention mid-regional pro-atrial natriuretic peptide (MR-proANP), a more reliable marker than ANP because of its longer half-life, although evidence supporting its use remains scarce [10,11].

## 2. Diagnosing HF

**Figure 1:**
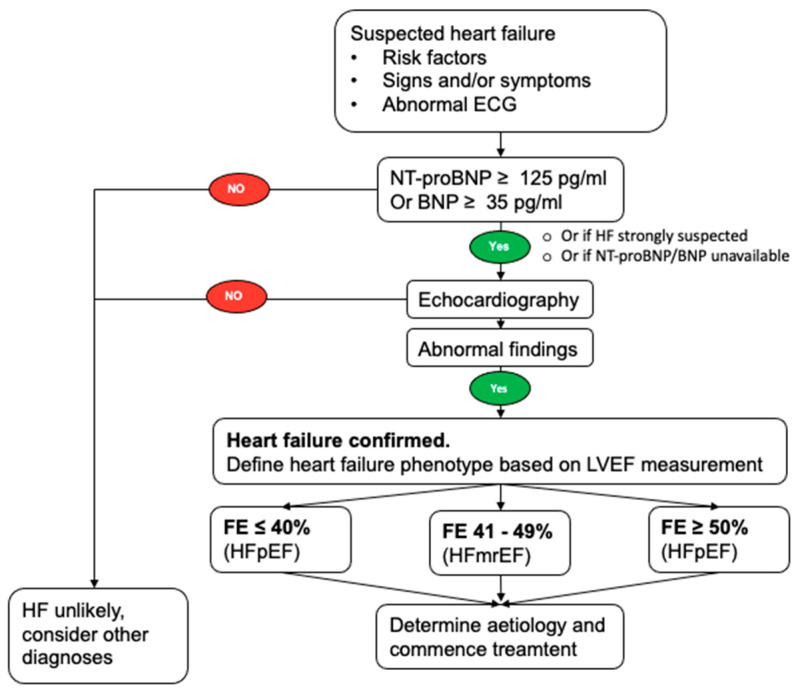
The diagnostic algorithm for heart failure. BNP: B-type natriuretic peptide; ECG: electrocardiogram; HFmrEF: heart failure with mildly reduced ejection fraction; HFpEF: heart failure with preserved ejection fraction; HFrEF: heart failure with reduced ejection fraction; LVEF: left ventricular ejection fraction; NT-proBNP: N-terminal pro-B type natriuretic peptide.

The diagnosis of HF relies on identifying signs and symptoms suggestive of HF (including dyspnea, orthopnea, fatigue, and peripheral edema) alongside a pertinent medical history indicating HF risk factors (history of myocardial infarction, hypertension, diabetes mellitus, chronic coronary artery disease). NP assessment, along with electrocardiography, is recommended at the onset of HF diagnostic evaluation (Figure 1).

Echocardiography constitutes the definitive diagnostic tool, allowing for the classification of HF into distinct subgroups based on left ventricular ejection fraction (Table 2). The diagnostic approach varies depending on the presentation of HF.

### 2.1. Acute Heart Failure

In the emergency setting, diagnostic procedures typically involve readily available tests such as electrocardiogram and echocardiography. However, NP testing (alongside other blood tests, including troponins, electrolytes, renal, liver, and thyroid function tests) is recommended. Low NP values (BNP < 100 pg/mL, NT-proBNP < 300 pg/mL, and MR-proANP < 120 pg/mL) facilitate ruling out acute heart failure (AHF) with high negative predictive value (NPV) [8]. Since BNP and NT-proBNP values have high sensitivity but low specificity, NP assessment has always been recommended, mainly to exclude rather than confirm HF. Nevertheless, a clinical consensus statement from the ESC Heart Failure Association in 2023 proposed age-specific NT-proBNP cutoffs for diagnosing HF: NT-proBNP ≥ 450 pg/mL for patients under 50 years; NT-proBNP ≥ 900 pg/mL for those aged 50 to 75 years; and NT-proBNP ≥ 1800 pg/mL for patients over 75 years [12]. Additionally, consideration should be given to alternative causes of dyspnea (such as pneumonia), and diagnosis should not be based exclusively on elevated BNP or NT-proBNP levels.

### 2.2. Chronic Heart Failure

In an outpatient setting, the NP assay can be useful in clarifying diagnostic uncertainty. Often, patients presenting to the emergency department with a new diagnosis of HF had previously reported nonspecific symptoms to their primary care physician, where NP assessment could have guided an earlier diagnosis and potentially prevented hospitalization. To address this issue, the 2023 ESC consensus document recommends utilizing a mnemonic acronym, the FIND-HF (fatigue, increased water accumulation, NP testing, and dyspnea–heart failure) to prompt NT-proBNP assessment for early HF diagnosis, preceding the onset of signs, such as lower-extremity edema, lung crackles, and jugular congestion [12].

The 2021 ESC guidelines established upper limits of normality specifically for this context—35 pg/mL for BNP and 125 pg/mL for NT-proBNP—demonstrating a high NPV ranging from 0.94 to 0.98. Although fewer data are available for MR-proANP, generally a value < 40 pmol/L excludes HF [8]. Also in this setting, the 2023 ESC consensus proposes a diagnostic algorithm incorporating age-specific NT-proBNP reference values—NT-proBNP ≥ 125 pg/mL for those younger than 50 years of age, NT-proBNP ≥ 250 pg/mL for ages 50 to 75 years, and NT-proBNP ≥ 500 pg/mL for those older than 75 years—indicating probable HF diagnosis necessitating echocardiography within 6 weeks (Table 3).

While this approach simplifies diagnosis by age-based NT-proBNP cutoffs, more complex algorithms considering sex, body mass index (BMI), atrial fibrillation (AF), and renal function have also been proposed (Table 4). Nonetheless, modified NT-proBNP reference values based on these variables remain expert opinion-derived and lack robust validation. Therefore, further studies are necessary to determine which of the two approaches (simplified or complex) can lead to a reduction in unnecessary procedures while minimizing resource waste as much as possible [12]. Changes in reference values of NT-proBNP suggested by the ESC 2023 consensus document based on variations in eGFR and BMI are listed in Table 4.

### 2.3. Heart Failure with Preserved Ejection Fraction (HFpEF)

HF is functionally classified (Table 1) and based on the extent of ejection fraction detected via echocardiogram (Table 2). The latter distinction is crucial for clinical–therapeutic management. Specifically, HF is categorized as follows: reduced ejection fraction (heart failure with reduced ejection fraction, HFrEF) when the ejection fraction (EF) is ≤40%, preserved ejection fraction (heart failure with preserved ejection fraction, HFpEF) if EF ≥ 50%, and mildly reduced ejection fraction (heart failure with mid-range ejection fraction, HFmrEF) if 40% < EF < 50%.

For diagnosing HFpEF, it is essential to identify structural, functional, or biochemical abnormalities suggesting the presence of diastolic dysfunction or increased filling pressures, both related to the left ventricle. Increased NPs constitute one of the diagnostic criteria mentioned above [13]. Notably, higher cutoff values have been distinguished in the presence of AF, a condition determining an increase in NPs. Consequently, diagnostic values are as follows: in sinus rhythm, BNP > 35 pg/mL or NT-proBNP > 125 pg/mL; if AF is present, BNP > 105 pg/mL and NT-proBNP > 365 pg/mL [8].

It is important to note that approximately 20% of patients with HFpEF exhibit nondiagnostic NP values, especially in cases of obesity. The diagnostic accuracy of NPs in confirming the presence of HFpEF is comparable to that of HFrEF, although values are generally higher in cases of reduced ejection fraction. Nonetheless, currently, there are no established thresholds for distinguishing between the two conditions [14,15].

## 3. Prognosis

In patients with HF, elevated BNP and NT-proBNP values are associated with an increased risk of major cardiovascular events, cardiovascular death, and all-cause mortality in both the long and short term. Therefore, guidelines recommend assessing biomarkers for risk stratification in patients with HF, aiming for optimal disease management over time [9]. Nevertheless, NP measurement is not recommended for certain patients, particularly those with advanced HF and poor prognosis or those with consistently elevated NP levels. It is crucial to differentiate the use of NPs between inpatient and outpatient settings.

### 3.1. Hospital Setting

In a patient admitted for HF, it is recommended to evaluate NPs at admission and shortly before discharge [9]. The ADHERE registry demonstrated in patients with acute decompensated HF (both with reduced and preserved ejection fraction) a nearly linear correlation between elevated BNP levels at admission and in-hospital mortality, independently of other clinical and laboratory variables [16].

Similarly, NT-proBNP is also a strong prognostic factor, and its evaluation at the beginning of a hospitalization for HF is equally recommended as BNP, as shown by the ProBNP Investigation of Dyspnea in the Emergency Department (PRIDE) study and an extensive meta-analysis, the International Collaborative of NT-proBNP Study [17,18].

Reassessment of NP levels before discharge is recommended due to their strong predictive value for death and HF rehospitalization [19,20,21]. According to Logeart et al., the pre-discharge BNP value is an important prognostic factor, surpassing clinical and echocardiographic parameters and even changes in BNP levels during hospitalization [22]. Therefore, NPs may potentially be useful in assessing therapeutic efficacy and discharge planning, but further studies are required to define the clinical implications of these observations. Indeed, currently, there is no demonstrated existence of therapeutic targets or a specific magnitude of reduction in peptide levels during hospitalization that correlates with improved outcomes [23].

### 3.2. Outpatient Setting

The guidelines recommend serial outpatient management for patients with chronic HF, even if they are stable and have good symptom control. Specifically, follow-up should include a check-up every 6 months in order to optimize therapy and detect asymptomatic progression of HF and its comorbidities. More frequent follow-up is advised after discharge or following changes in therapy. According to the ESC guidelines, annual ECGs are recommended, and the echocardiograms should be reserved for instances of changes in the clinical presentation or therapy [8]. American guidelines emphasize the utility of NPs in the follow-up of the patient with chronic HF, recommending their measurement to achieve appropriate risk stratification. Indeed, there is substantial evidence supporting the prognostic value of NPs in the management of chronic HF [17,24,25,26,27,28].

A systematic literature review examined 19 studies investigating the relationship between BNP levels and the risk of major cardiovascular events or death, demonstrating that every 100 pg/mL increase in BNP was associated with a 35% increase in the relative risk of death, highlighting BNP as a robust prognostic indicator [29].

In a population of 85 patients with chronic HF, BNP values < 73 pg/mL were significantly associated with higher survival rates compared to higher BNP levels [30]. Similarly, in another study involving 102 patients with class III and IV HF, persistently elevated BNP levels (>240 pg/mL) after treatment had a sensitivity of 73% and a specificity of 74% in predicting 2-year mortality [31]. NPs have the potential to serve as a surrogate for the functional status of a patient with HF. Therefore, measuring BNP and NT-proBNP could be a useful and cost-effective screening tool for specialists in disease management, reducing the need for additional, more expensive cardiac testing [26].

## 4. Heart Failure Therapy Optimization

Studies showed that a reduction in BNP or NT-proBNP levels following drug treatment for HF, optimized according to guidelines, leads to a more favorable clinical outcome compared to stability or elevation in NPs [31,32,33,34]. NPs assume prognostic value, prompting consideration of their use in therapy management. Several clinical trials investigated the use of biomarkers, particularly BNP and/or NT-proBNP, to guide drug therapy in HFrEF, but results are inconsistent [35,36,37,38,39,40].

While evidence supports their use as prognostic markers, it remains uncertain whether biomarker-guided therapy offers additional benefits over guideline-based approaches. Therefore, routine measurement of BNP and/or NT-proBNP for therapy management is not currently supported. This aspect is of great importance in everyday clinical practice, and further evidence is needed before specific recommendations on whether and how to modify treatment according to changes in natriuretic peptides.

## 5. Risk Stratification in Patients at Risk for HF

Prevention of HF through proper identification of individuals at risk is crucial. The American guidelines recognize the importance of prevention by defining stage A HF, which includes asymptomatic individuals without functional or structural cardiac changes, but with conditions such as diabetes mellitus, hypertension, or vascular disease (Table 5). NP measurement may serve as a potential screening tool in this context. Specifically, in stage A patients, evaluation of BNP or NT-proBNP is recommended for management by a multidisciplinary team, including cardiovascular specialists, to prevent the development of left ventricular dysfunction and HF. Evidence supporting the use of NPs in the preventive setting is substantial [41]. For instance, in a large-scale, single-center clinical trial, the STOP-HF (St Vincent’s Screening to Prevent Heart Failure) trial, 697 individuals at risk of HF underwent BNP assessment, echocardiogram, specialist follow-up, and potentiation of therapy with SRAA antagonists in cases of BNP > 50 pg/mL. BNP-based screening showed a reduction in the incidence of left ventricular dysfunction and HF compared to controls [42]. NT-proBNP measurement appears to predict the risk of death and major cardiovascular events even better than other traditional biomarkers like C-reactive protein [43,44].

More recently, the ESC Heart Failure Association consensus document further distinguished patients at risk of HF into two categories: heart health or heart stress, based on the absence or presence, respectively, of increased NP values in asymptomatic patients with risk factors for HF. The novelty of this consensus paper is the proposal of a diagnostic algorithm for preventing HF in these individuals. Cutoff values are suggested based on studies in patients with diabetes, given the abundant evidence available in this population. Notably, an NT-proBNP value < 50 pg/mL denotes a heart health condition, excluding cardiac pathology. For heart stress diagnosis, age-specific reference values are suggested: NT-proBNP > 75 pg/mL for patients younger than 50 years; NT-proBNP > 150 pg/mL for ages 50 to 74 years; and for those older than 75 years, NT-proBNP > 300 pg/mL. Elevated NT-proBNP concentrations suggest the likelihood of cardiac stress, leading to further investigations such as echocardiography, considering the possible presence of AF and chronic kidney disease. Heart stress patients should be educated about a healthy lifestyle, optimizing hypertension, diabetes, and hypolipidemic treatment. Follow-up includes reassessment of NT-proBNP within the next 6–12 months to assess the response to any intervention (Table 6) [12].

These recommendations underscore the importance of cardiovascular risk stratification in guiding therapeutic choices and optimizing the management of patients at risk, ultimately leading to improved outcomes and reduced health-care expenditures. However, further clinical studies are needed to validate these recommendations derived from consensus documents and provide additional evidence in the future.

## 6. The Diabetologist’s Point of View

Diabetes can contribute to the development of structural heart disease and HF through systemic, myocardial, and cellular mechanisms. Thereby, glucose metabolism abnormalities are a known cardiovascular risk factor. At the same time, HF can be considered a risk factor for diabetes, as metabolic impairment is intrinsic to HF pathophysiology, with insulin resistance observed in up to 60% of patients with HF [45]. The concurrence of HF and diabetes involves worse clinical outcomes compared to HF alone. Population-based studies show that concomitant diabetes increases the risk of death in both hospitalized and ambulatory HF patients. Additionally, multivariable HF risk models (e.g., the MAGGIC [Meta-Analysis Global Group in Chronic Heart Failure] risk score [46]), identify diabetes as an independent risk factor for mortality. Diabetes also worsens non-mortality outcomes, including a 50% higher risk of hospitalization, modestly increased readmission rates, and reduced health-related quality of life in patients with both HF and diabetes [47].

It is in this context that the interplay between HF and diabetes becomes particularly relevant, and according to the ACC/AHA/HFSA guidelines, all patients with diabetes mellitus are classified as stage A HF. To date, with antidiabetic drugs capable of providing cardiovascular protection, diabetologists must increasingly consider the importance of proper risk stratification [48,49]. The American Diabetes Association (ADA) recently published a consensus document highlighting the high HF prevalence in patients with diabetes (up to 22%), aiming to guide diabetologists in the optimal screening and diagnosis of HF in the patient with diabetes [50]. The ADA guidelines recommend using the ACC/AHA’s ASCVD risk calculator to stratify cardiovascular risk in patients with diabetes. However, the performance of this calculator remains suboptimal, underscoring the need for new biomarker predictors [51].

### 6.1. The Utility of Natriuretic Peptides

As previously described, several studies support the ability of NPs to predict cardiovascular risk in diabetic patients. In a population of 1690 individuals with type 2 diabetes mellitus, NT-proBNP alone was shown to predict the risk of cardiovascular adverse events even better than other traditional risk calculation models [52]. The predictive value of NPs was demonstrated to be independent of glycemic status [53]. A population-based study of 5502 individuals without HF, but with different glycemic status (3380 normoglycemic, 1125 with prediabetes, and 997 with diabetes) showed that elevated NT-proBNP values (>100 pg/mL) were associated with all-cause and cardiovascular mortality, regardless of glycemic levels, age, sex, body mass index, or other cardiovascular risk factors [54].

Clinical benefits of implementing NT-proBNP screening were demonstrated by a small, randomized, single-center study, the PONTIAC (NT-proBNP Guided Primary Prevention of CV Events in DIABETIC PATIENTs) trial in which patients with diabetes in primary cardiovascular prevention with NT-proBNP > 125 pg/mL underwent therapy intensification with SRAA antagonists and/or beta-blockers, achieving a significant reduction in cardiovascular-related death and hospitalizations [55].

These studies corroborate the evidence supporting the use of NT-proBNP measurement for risk stratification in diabetic patients and guidance of medical treatment. The diagnostic algorithm proposed by the ESC consensus document published in 2023 (Table 6) is based precisely on the evaluation of NT-proBNP reference values derived from studies in patients with diabetes. In clinical practice, diabetologists often need to add additional antidiabetic drugs for patients in primary cardiovascular prevention. The proposed algorithm should ensure a more informed therapeutic choice, favoring certain classes of antidiabetic drugs protective from cardiovascular risk [51]. Other potentially useful biomarkers for risk stratification include the Fibrosis-4 index (FIB-4), which estimates the risk of liver fibrosis based on transaminase levels and platelet count. Both FIB-4 and NT-proBNP have been shown to be independently associated with cardiovascular mortality and all-cause mortality in patients with metabolic (dysfunction)-associated steatotic liver disease, a condition frequently associated with type 2 diabetes mellitus, suggesting that their combined use may provide a useful risk stratification tool in clinical practice [56]. However, further large-scale randomized controlled trials are needed to verify the actual clinical benefits of the abovementioned preventive interventions.

### 6.2. Sodium Glucose Transporter 2 Inhibitors (SGLT2-Is) and Natriuretic Peptides

Although initially developed as antidiabetic drugs, SGLT2-Is have demonstrated significant cardiovascular and renal protective effects [57]. Meta-analyses of cardiovascular outcomes from randomized clinical trials of SGLT2i showed a 31% (HR 0.69, 95% CI 0.61–0.79, *p* < 0.001) reduction in HF hospitalizations in both primary and secondary cardiovascular prevention subjects. This finding becomes more significant when considering the low number of subjects needed to treat to prevent hospitalizations due to decompensation of 100 (79–147) over a mean follow-up period of 3.3 years, irrespective of age, sex, BMI, eGFR, and baseline drug therapy. Real-life studies have also confirmed the benefits of SGLT2-is [58]. Therefore, guidelines recommend the use of SGLT2-is in patients with known HF (both with preserved or reduced EF) or in secondary cardiovascular prevention [59,60]. In this context, the influence of SGLT2-is on PNs was investigated. The effect of dapagliflozin was examined in the DEFINE study, where no significant difference in mean NT-proBNP values was observed at 12 weeks of dapagliflozin treatment compared to placebo. However, the authors noted a greater baseline variability in NT-proBNP, precluding the analysis of mean NT-proBNP and thus hindering the identification of subtle biomarker changes. Regardless, a higher proportion of patients treated with dapagliflozin experienced a ≥20% reduction in NT-proBNP [61]. Conversely, in the EMPEROR-Reduced study, empagliflozin significantly reduced NT-proBNP levels at all examined time points alongside decreasing the risk of major cardio-renal events, regardless of baseline NT-proBNP concentration. Notably, at 52 weeks of follow-up, the mean reduction in NT-proBNP compared to the placebo group was 13% (*p* < 0.001). Additionally, NT-proBNP was confirmed to have prognostic value in the intervention group, with lower NT-proBNP levels associated with a lower risk of subsequent cardiovascular death or HF hospitalization, demonstrating how NT-proBNP concentration after empagliflozin introduction better predicts prognosis than NT-proBNP value at baseline [62].

## 7. Conditions of Reduced Reliability of Natriuretic Peptides

It is important to recognize several factors that can affect the reliability of NPs, independently of cardiac injury (Table 7). The most common conditions that can alter NP values are discussed below [8,9].

### 7.1. Chronic Kidney Disease

The elimination of BNP involves its binding to neprilysin and glomerular filtration, while NT-proBNP is eliminated solely through the renal system. This results, when glomerular filtration rate (eGFR) is reduced, in increased values of BNP and to a greater extent NT-proBNP. Therefore, in patients with renal failure, NPs are less reliable as markers of HF [63], necessitating appropriate diagnostic cutoffs. In the aforementioned PRIDE study, the adoption of higher diagnostic values based on glomerular filtration rate and patient age resulted in satisfactory levels of sensitivity and specificity [64,65]. The ESC Heart Failure Association consensus document suggests modifications of NT-proBNP reference values based on eGFR reduction (Table 4) [12]. However, further studies are required to validate these recommendations.

### 7.2. Age, Sex, and Body Mass Index

Obese patients may exhibit lower BNP and NT-proBNP values than non-obese individuals, reducing their diagnostic sensitivity. However, higher BNP values within each BMI category are associated with worse outcomes, retaining their prognostic value [66]. The ESC Heart Failure Association consensus document provides guidance on interpreting NT-proBNP based on BMI (Table 4) [12]. Additionally, older age and female sex are associated with higher NP values.

### 7.3. Effects of Sacubitril–Valsartan Therapy

In patients with HF receiving sacubitril–valsartan therapy, plasma BNP levels may become less reliable due to the inhibition of neprilysin, the enzyme responsible for BNP degradation. Conversely, NT-proBNP is not degraded by neprilysin, decreasing consensually with ARNI use more than BNP, as demonstrated in PIONEER-HF and PARADIGM-HF. Thus, NT-proBNP levels remain reliable and can be used to assess disease progression [67,68].

### 7.4. Right Heart Failure

Elevated BNP and NT-proBNP levels may be misinterpreted if right HF is solely secondary to pulmonary disease, such as pulmonary embolism or pulmonary arterial hypertension.

### 7.5. Impact of Genetic Variation on Interindividual Variability

Interindividual variability is considerable. In a systematic review that included 558 patients with chronic HF undergoing outpatient follow-up, 24% of 449 symptomatic patients had BNP values < 100 pg/mL, while some of the 109 asymptomatic patients showed an increase in plasma BNP, up to values as high as 572 pg/mL [69]. Genetic variations can affect NP function and related disease states. Several studies have identified different single-nucleotide polymorphisms (SNPs) in genes from which are produced NP precursor peptides: the natriuretic peptide precursor A (NPPA) and NPPB. Notably, one of the most studied SNPs is a variant in the promoter region, called rs198389. The latter has been shown to be associated with higher levels of BNP and its precursor NT-proBNP with clinical implication [70,71]. For instance, subsequent studies demonstrated that the minor allele of rs198389 was associated with decreased likelihood of ventricular dysfunction in patients following coronary artery bypass grafting, reduced readmission rates post-discharge, and a lower risk of developing future type 2 diabetes mellitus [72,73,74]. Similar research on minor alleles of SNPs in the NPPA gene has shown increased levels of ANP and BNP. Moreover, minor alleles of SNPs in the ANP gene have been associated with lower blood pressure among white individuals [75]. It should be emphasized that studies on NPPA polymorphisms were generally smaller and had more limited follow-up compared to those investigating NPPB gene SNPs.

Additionally, different NP receptor genotypes, among which NPRA is the NP system gene most studied, could influence clinical phenotype with repercussions on hypertension, cardiovascular state, and myocardial infarction [76].

Genetic testing may provide insight into the substantial variability observed both between and within individuals, potentially enabling a more personalized interpretation of BNP and NT-proBNP levels. Future research should focus on integrating genotype information into well-characterized clinical cohorts, including NP testing, to better assess clinical outcomes in patients with heart failure or coronary artery disease. Moreover, a deeper understanding of genetic variations may be useful in the future for optimizing heart failure therapy, particularly in improving the management of recombinant exogenous NP infusion and neprilysin inhibitor [77].

Currently, in clinical practice, serial measurements of BNP and NT-proBNP concentrations are useful for monitoring a patient’s clinical course, being more reliable than single measurements.

### 7.6. Laboratory Variability

Different tests may result in misinterpretation of the value of NPs, and it is necessary to consider this variability when interpreting serial results. The analytical variability for NT-proBNP is generally less than that for BNP [78,79].

## 8. Applications Other than Heart Failure

A disproportionately elevated NT-proBNP value relative to the degree of HF, along with persistently elevated troponin levels, should raise suspicion of cardiac amyloidosis and prompt further investigation [8].

## 9. Conclusions

NPs are valuable for diagnosing and prognosticating HF and may also be beneficial in the preventive setting. Patients with diabetes mellitus are inherently at risk of developing HF, making outpatient diabetes clinics suitable settings for using NPs for cardiovascular risk stratification. Regular assessment of NPs may facilitate early referral for diagnostic investigation and optimization of cardiovascular therapy, ultimately preventing and slowing the progression of HF.

## Figures and Tables

**Table 1:** Functional classification of heart failure according to the New York Heart Association based on symptom severity and physical activity.

Class	Patient Symptoms
**I**	No limitation of physical activity. Ordinary physical activity does not cause dyspnea, fatigue, or palpitation.
**II**	Slight limitation of physical activity. Comfortable at rest. Ordinary physical activity results in fatigue, palpitation, shortness of breath, or angina.
**III**	Marked limitation of physical activity. Comfortable at rest. Less than ordinary physical activity cause fatigue, palpitations, shortness of breath, or angina.
**IV**	Symptoms of heart failure at rest. Any physical activity causes further discomfort.

**Table 2:** Classification of heart failure based on left ventricular ejection fraction.

Type of HF		HFrEF	HFmrEF	HFpEF
Criteria	1	Symptoms ± signs ^a^ suggesting HF	Symptoms ± signs ^a^ suggesting HF	Symptoms ± signs ^a^ suggesting HF
	2	LVEF ≤ 40%	LVEF 41–49% ^b^	LVEF ≥ 50%
	3	–	–	Objective evidence of cardiac structural and/or functional abnormalities consistent with the presence of LV diastolic dysfunction/raised LV filling pressures, including raised natriuretic peptides

^a^ Signs may not be present in the early stages of HF (especially in HFpEF) or in optimally treated patients. ^b^ For a diagnosis of HFmrEF, the presence of other evidence of structural heart disease (e.g., increased left atrial size, LV hypertrophy, or echocardiographic measures of impaired LV filling) makes the diagnosis more likely. HF, heart failure; HFrEF, heart failure with reduced ejection fraction; HFmrEF, heart failure with mildly reduced ejection fraction; HFpEF; heart failure with preserved ejection fraction; LVEF, left ventricular ejection fraction.

**Table 3:** Diagnostic algorithm in patients with clinical suspicion of heart failure in outpatient setting.

HF Suspicion in Outpatient Setting (Medical History, Physical Exam, ECG)			
NT-proBNP (pg/mL)	Age (years)	Diagnosis ang management	
<125	Age-independent	HF very unlikely	Evaluation for non-cardiac cause advised
-	<50	HF not likely (gray zone). Consider BMI, AF, eGFR and treatment (diuretics, RASi, MRA)	Consider alternative diagnosis If clinical suspicion remains, arrange echocardiography
126–249	50–74		
126–499	≥74		
≥125	<50	HF likely	Treat as appropriate Arrange for echocardiography (≤6 weeks)
≥250	50–74		
≥500	≥74		
≥2000	Age-independent	HF very high risk	Priority echocardiography Evaluation by heart failure team (≤2 weeks)

HF, heart failure; ECG, electrocardiogram; NT-proBNP, N-terminal pro-B-type natriuretic peptide; BMI, body max index; AF, atrial fibrillation; eGFR, estimated glomerular filtration rate; MRA, mineralocorticoid receptor antagonist; RASi, renin–angiotensin system inhibitor.

**Table 4:** Changes in reference values of NT-proBNP suggested by the ESC 2023 consensus document based on variations in eGFR and BMI.

**eGFR (mL/min/1.73 m^2^)**	**Changes in reference values of NT-proBNP**
eGFR < 30	Increase of 35%
30 ≤ eGFR < 45	Increase of 25%
45 ≤ eGFR < 60	Increase of 15%
**BMI (kg/m^2^)**	**Changes in reference values of NT-proBNP**
30 ≤ BMI < 35	Decrease of 25%
35 ≤ BMI < 40	Decrease of 30%
BMI ≥ 40	Decrease of 40%

Abbreviations: eGFR, estimated glomerular filtration rate; NT-proBNP, N-terminal pro-B-type natriuretic peptide; BMI, body mass index [12].

**Table 5:** Stages of heart failure according to the 2022 ACC/AHA/HFSA guidelines.

Stages of HF	Definition and Criteria
**Stage A** **At risk of HF**	People who are at risk for HF, but without symptoms, structural or functional heart disease, or elevated NPs. Risk factors include: Hypertension Atherosclerotic CVD DM Obesity and metabolic syndrome Exposure to cardiotoxic agents Genetic variants for cardiomyopathy and family history of cardiomyopathy
**Stage B** **Pre-HF**	No symptoms or signs of HF and evidence of 1 of the following: Structural heart disease - Reduced left or right ventricular systolic function - Reduced ejection fraction, reduced strain - Ventricular hypertrophy - Chamber enlargement - Wall motion abnormalities - Valvular heart disease Evidence of increased filling pressures - By invasive hemodynamic measurements - By noninvasive imaging suggesting elevated filling pressures (e.g., Doppler echocardiography) Patients with risk factors and: - Increased levels of BNPs or - Persistently elevated cardiac troponin in the absence of competing diagnoses resulting in such biomarker elevations such as ACS, CKD, pulmonary embolus, or myopericarditis
**Stage C** **Symptomatic HF**	Structural heart disease with current or previous symptoms of HF
**Stage D** **Advanced HF**	Marked HF symptoms that interfere with daily life and with recurrent hospitalizations despite attempts to optimize GDMT.

HF, heart failure; NPs, natriuretic peptides; DM, diabetes mellitus; CVD, cardiovascular disease; ACS, acute coronary syndrome; CKD, chronic kidney disease; BNP, B-type natriuretic peptide; GDMT, guideline-directed medical therapy [9].

**Table 6:** Diagnostic algorithm in healthy patients at risk of heart failure.

Screening for Heart Stress in Asymptomatic Patients with T2DM (or Other CVRFs)			
NT-proBNP (pg/mL)	Age (years)	Diagnosis and management	
≤50	Age-independent	Heart stress very unlikely	Repeat NT-proBNP in one year
51–74	<50	Heart stress not likely (Grey zone)	Repeat NT-proBNP in six months
51–149	50–74		
51–299	≥74		
≥75	<50	Heart stress likely	Arrange echocardiography Assessment by heart failure team if cardiac dysfunction present Reassess NT-proBNP every 6–12 months
≥150	50–74		
≥300	≥74		

T2DM, type 2 diabetes mellitus; CVRFs, cardiovascular risk factors; NT-proBNP, N-terminal pro-B-type natriuretic peptide; HF, heart failure.

**Table 7:** Conditions that increase BNP and NT-proBNP levels.

Cardiac Conditions	Non Cardiac Conditions
Right ventricular failure	Advanced age
Acute coronary syndrome	Anemia
Left and/or right ventricular hypertrophy	Renal impairment
Pericarditis	Severe burns
Myocarditis	Pulmonary embolism
Hypertrophic or restrictive cardiomyopathy	Ischemic stroke
Valvulopathies	Subarachnoid hemorrhage
Congenital cardiac abnormalities	Hepatic dysfunction (liver cirrhosis with ascites)
Atrial and ventricular tachyarrhythmias	Pulmonary arterial hypertension
Cardiac tamponade	Paraneoplastic syndrome
Cardioversion, shock delivered by ICD	COPD, OSAS
Cardiac surgery	Severe infections (bacterial sepsis, pneumonia
Myocardial toxic–metabolic insults, including cancer chemotherapy	Severe endocrine–metabolic abnormalities (diabetic ketoacidosis, thyrotoxicosis)

ICD: implantable cardioverter–defibrillator; COPD: chronic obstructive pulmonary disease; OSAS: obstructive sleep apnea syndrome.

## Data Availability

No original data were generated for the present manuscript.
